# Preexisting mild cognitive impairment as a risk factor for COVID-19 infection: A scoping review and case reports

**DOI:** 10.1192/j.eurpsy.2022.1267

**Published:** 2022-09-01

**Authors:** P. Catapano, M. Messina, A. Russo, C. Tucci, M. Luciano, V. De Santis, F. Perris, F. Catapano, N. Coppola, M. Fabrazzo

**Affiliations:** 1 Università degli studi della Campania “Luigi Vanvitelli”, Dipartimento Di Psichiatria, Napoli, Italy; 2 Università degli studi della Campania “Luigi Vanvitelli”, Dipartimento Di Salute Mentale E Fisica E Medicina Preventiva, Napoli, Italy; 3 University of Campania “Luigi Vanvitelli”, Department Of Psychiatry, Naples, Italy

**Keywords:** Covid-19, delirium, SARS-Co-V2, mild cognitive impairment

## Abstract

**Introduction:**

SARS-Co-V2 neuroinvasive ability might be the basis for the onset of delirium and neuropsychiatric outcomes.

**Objectives:**

We hypothesized that some infected patients with preexisting cognitive dysfunction may present delirium as unique manifestation of COVID-19 infection or as a prodrome of a new episode consistent with the psychiatric history.

**Methods:**

We conducted a PubMed literature search to verify whether cognitive impairment might predispose to COVID-19. We included three patients with mild cognitive impairment and delirium at admission for SARS-Co-V2 suspected infection. Delirium was diagnosed according to DSM-5 criteria‚ Cognitive Assessment Method and Coma Glasgow Scale.

**Results:**

Literature analysis evidenced patients presenting delirium or delirium-like symptoms as clinical manifestation of COVID-19, plus a cognitive impairment‚ from mild to severe‚ which preexisted or was evidenced during the acute phase or after the infection. Most studies described delirium in patients with a past neurological/psychiatric history. Contrasting data emerged on the potential link between COVID-19 and delirium in patients with cognitive impairment and without a past neuropsychiatric history. Our patients had no history of other medical complications. Our first patient had no psychiatric history‚ the second reported only a depressive episode‚ and the third had story of bipolar disorder. Delirium resolved completely after 2 days in the first patient. The other patients required 4 and 14 days to resolve: delirium appeared as the prodrome of a new psychiatric episode in line with their past history.

**Conclusions:**

Clinicians should acknowledge the possibility that COVID-19 infection may appear as delirium and acute psychiatric sequelae as unique manifestation.

**Disclosure:**

No significant relationships.

